# Diagnosis, Treatment, and Prevention of Ileostomy Complications: An Updated Review

**DOI:** 10.7759/cureus.34289

**Published:** 2023-01-27

**Authors:** Shingo Tsujinaka, Hideyuki Suzuki, Tomoya Miura, Yoshihiro Sato, Hiroko Murata, Yasue Endo, Kyoko Hoshi, Yoshie Sato, Chikashi Shibata

**Affiliations:** 1 Gastroenterological Surgery, Tohoku Medical and Pharmaceutical University, Sendai, JPN; 2 Nursing, Tohoku Medical and Pharmaceutical University Hospital, Sendai, JPN

**Keywords:** ileostomy, end ileostomy, diverting stoma, covering ileostomy, complications

## Abstract

An ileostomy is associated with multiple complications that may frequently or persistently affect the life of ostomates. All healthcare professionals should have knowledge of the diagnosis, treatment, and prevention of ileostomy complications. Peristomal dermatitis is caused by watery and highly alkaline effluent. Skin protective products are typically used for local treatment. Ischemia/necrosis occurs due to insufficient arterial blood supply. Retraction is seen in patients with a bulky mesentery and occurs following ischemia. Convex stoma appliances can be used for skin protection against fecal leakage. Small bowel obstruction (SBO) is common and occurs only at the stoma site. Trans-stomal decompression is most effective in these cases. High output stoma (HOS) is defined as a condition when the output exceeds 1,000*-* 2,000 ml/day, lasting for one to three days. Treatment includes intravenous fluid and electrolyte resuscitation followed by restriction of hypotonic fluid and the use of antimotility (and antisecretory) drugs. Stomal prolapse is a full-thickness protrusion of an inverted bowel. Manual reduction is attempted initially, whereas emergency bowel resection may be needed for incarcerated cases. A parastomal hernia (PSH) is an incisional hernia of the stoma site. Surgery is considered in cases of incarceration, but most cases are manageable with non-surgical treatment.

## Introduction and background

An ileostomy is performed in either an end or loop fashion based on the pathology that necessitated stoma creation. An end ileostomy is indicated when the stoma becomes the most distal part after a large area of the bowel is resected, or no reconstructive procedure was made for the bowel continuity (e.g., "Brooke ileostomy" after proctocolectomy for inflammatory bowel diseases). Diverting stoma (DI) is essentially created with a loop formation, which protects the anastomosis from leakage and facilitates healing in patients undergoing total mesorectal excision for rectal cancer or restorative proctocolectomy with ileal pouch-anal anastomosis for ulcerative colitis. DI has been preferred over diverting colostomy (DC) due to a lower incidence of surgical site infections, stomal prolapse, retraction, parastomal hernia, and incisional hernias [1]. Further, DI is advantageous compared to DC in terms of a technically easier stoma reversal. However, ileostomy is associated with stoma-related complications such as dehydration or peristomal dermatitis, which may repeatedly or persistently affect the life of ostomates [1, 2]. Therefore, surgeons, nurses, and all stoma care providers should have knowledge about the diagnosis, treatment, and prevention of ileostomy complications to maintain the best quality of care and mitigate the consequences of complications.

## Review

Methods

An electronic English literature search was performed using the PubMed/MEDLINE database for articles published from the inception to November 30th, 2022. The search terms included "end ileostomy", "diverting ileostomy", or "covering ileostomy", and "complications". Inclusion criteria for the article type were systematic reviews and meta-analyses, randomized controlled trials, retrospective observational studies, and narrative reviews focused on nursing practice. Case reports, editorials, and letters to the editor were not included.

Ileostomy complications

Peristomal dermatitis

**Figure 1:**
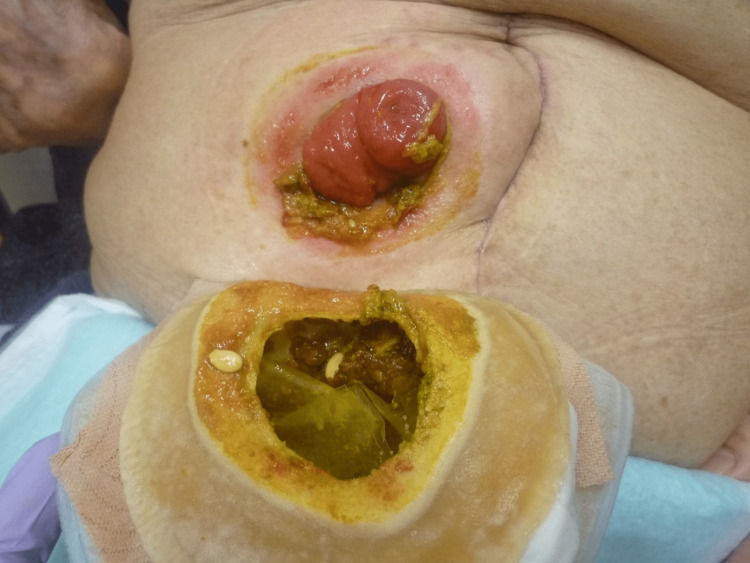
Peristomal dermatitis with stool and the stoma appliance Peristomal skin is irritated by ileostomy output which is watery, highly alkaline and contains proteolytic enzymes. Image created by the authors

**Figure 2:**
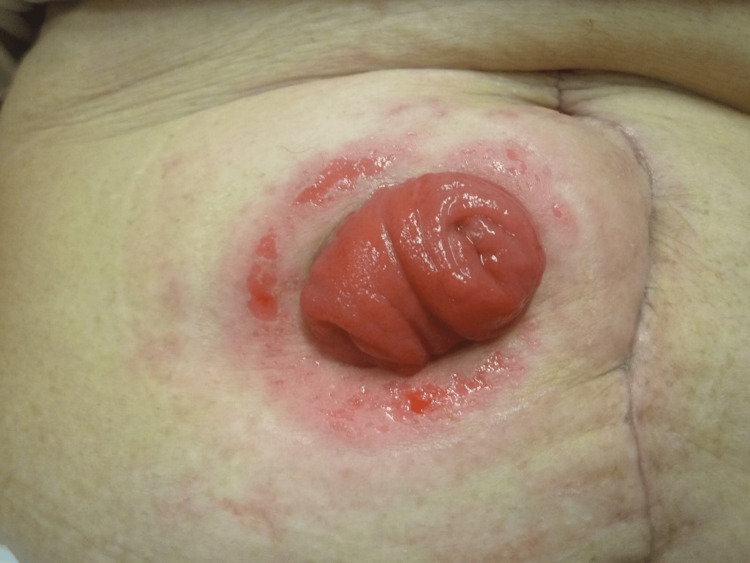
After removing stool and the stoma appliance Peristomal skin is reddened and eroded. Image created by the authors

The skin complication associated with ileostomy is peristomal dermatitis. The output from an ileostomy is watery, highly alkaline, and contains proteolytic enzymes that constantly irritate the peristomal skin [3]. Exposure to the effluent causes loss of epidermal integrity and maceration, which leads to painful ulceration [3, 4] (Figures 1, 2). Barrier rings/seals, skin barrier powder, paste, and paste strips are useful for local treatment [5]. Convex stoma appliances can be used for those with low stoma height. The best method to prevent peristomal dermatitis is to create a properly protruding ileostomy with a 2-3 cm height [3, 4].

Ischemia/necrosis

**Figure 3:**
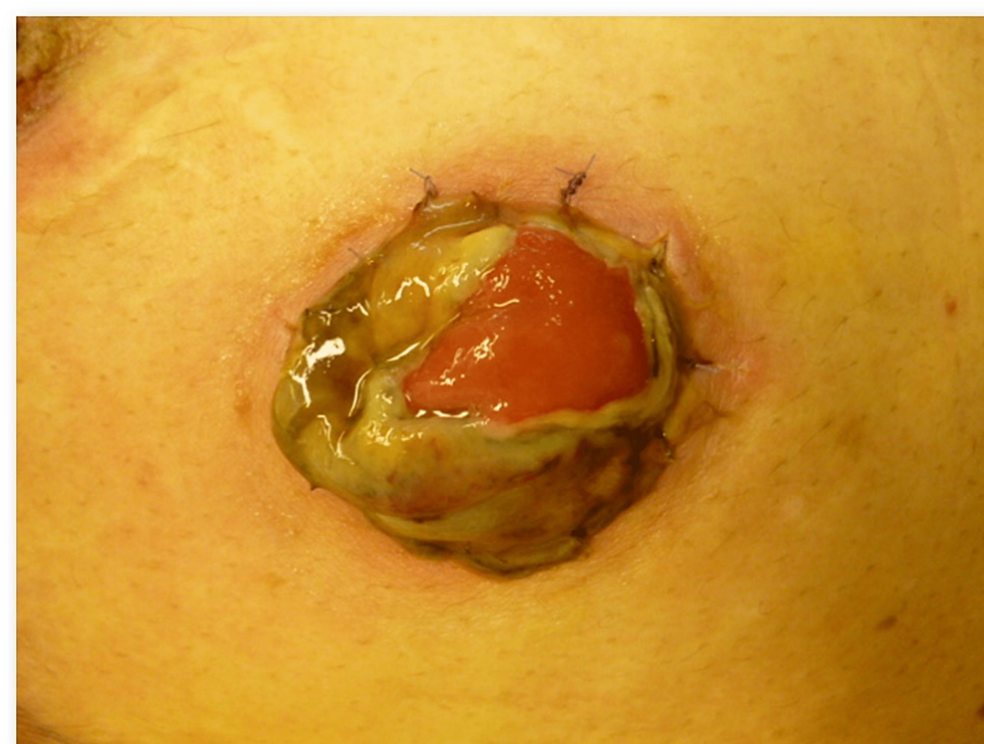
Ischemia and necrosis Mucosal ischemia typically begins at the mucocutaneous junction. Image created by the authors

Ischemia/necrosis typically occurs within 24 hours after stoma construction and begins with discoloration of the mucocutaneous junction at the site (Figure 3). A newly constructed stoma appears darkish and edematous with early venous congestion in the mesentery, which must be distinguished from the insufficient arterial blood supply when the edema subsides. Ileostomy carries a very low risk of necrosis because of its dense vascular network, and its incidence is reportedly as low as 1-5% [4]. If ischemia/necrosis is superficial within a subcutaneous layer, a 'watch and wait' policy may be effective, but mucocutaneous separation may occur. If it is limited above the fascia, immediate stomal revision may not be needed; however, care must be taken for delayed progression of stenosis. If it extends deeper below the fascia, emergency reconstruction should be performed. Avoiding injury to the peristomal vessels and tension in the mesentery are crucial preventive measures at the time of stoma creation.

Retraction

**Figure 4:**
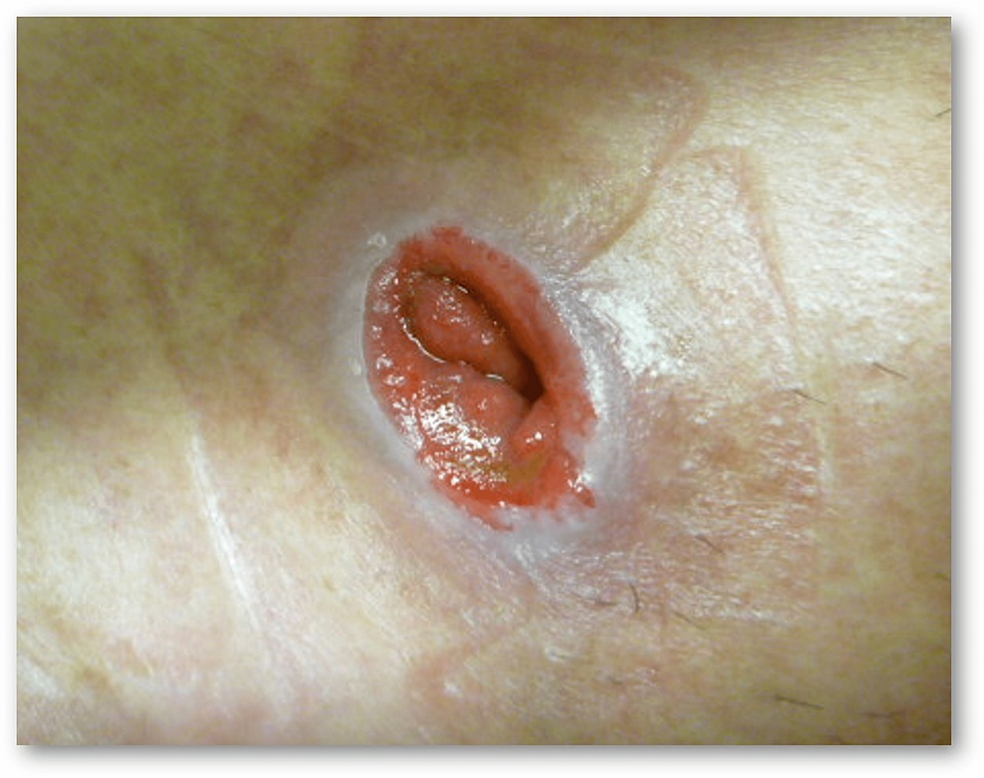
Retraction The opening of the stoma is under the skin level. Image created by the authors

The stoma appears depressed at or under the skin level when the bowel is pulled invertedly towards the abdominal wall (Figure 4). This is termed retraction, and its reported incidence is 2.9-17%, with a decreasing trend in recent years [4, 6, 7]. The most common risk factor is tension in the mesentery. Retraction is also frequently seen in patients with high body mass index, in whom fat mesentery and well-developed panniculus cause a 'downforce effect' to the mucocutaneous junction.

Convex stoma appliances can be used to decrease the severity of skin damage caused by fecal leakage. Stoma rods have traditionally been used; however, recent studies have shown that rods do not reduce the incidence of retraction but increase adverse events, including edema, abscess, bleeding, necrosis, and dermatitis [6, 8]. Stomal revision is the definitive treatment for this condition. Prevention involves obtaining an adequate length of the mesentery with a good blood supply.

Small bowel obstruction

**Figure 5:**
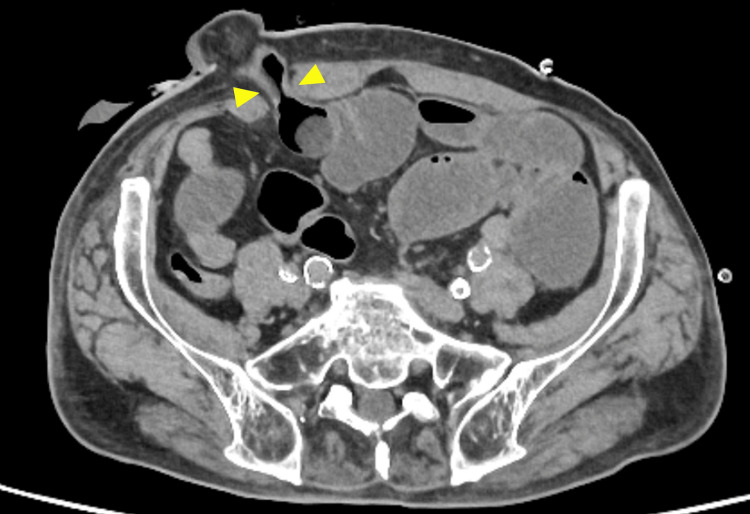
CT image of a small bowel obstruction at the stoma site (axial view) The arrowheads indicate a luminal narrowing of the stoma limb causing small bowel obstruction. Image created by the authors

**Figure 6:**
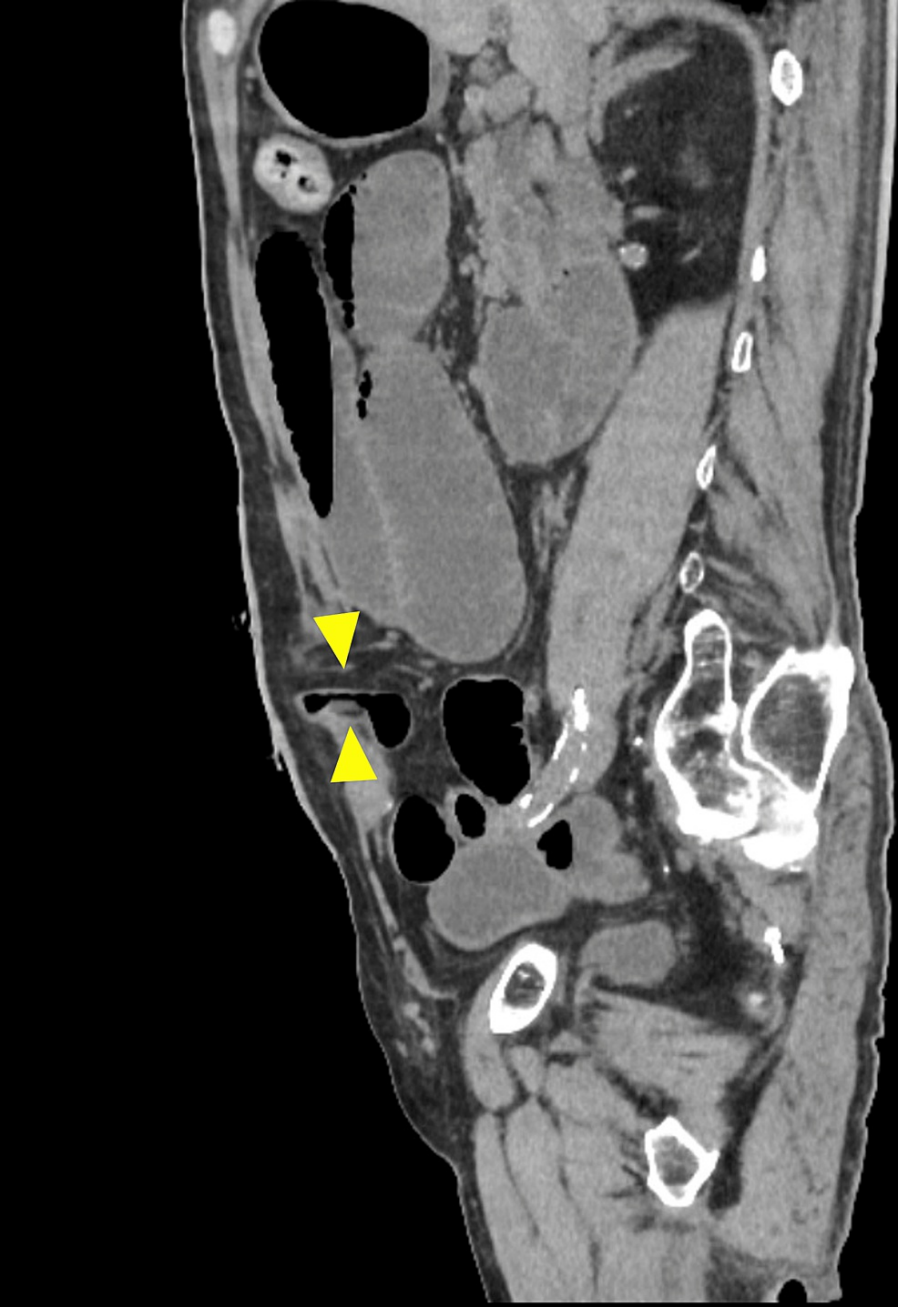
CT image of a small bowel obstruction at the stoma site (sagittal view) The arrowheads indicate a luminal narrowing of the stoma limb causing small bowel obstruction. Image created by the authors

**Figure 7:**
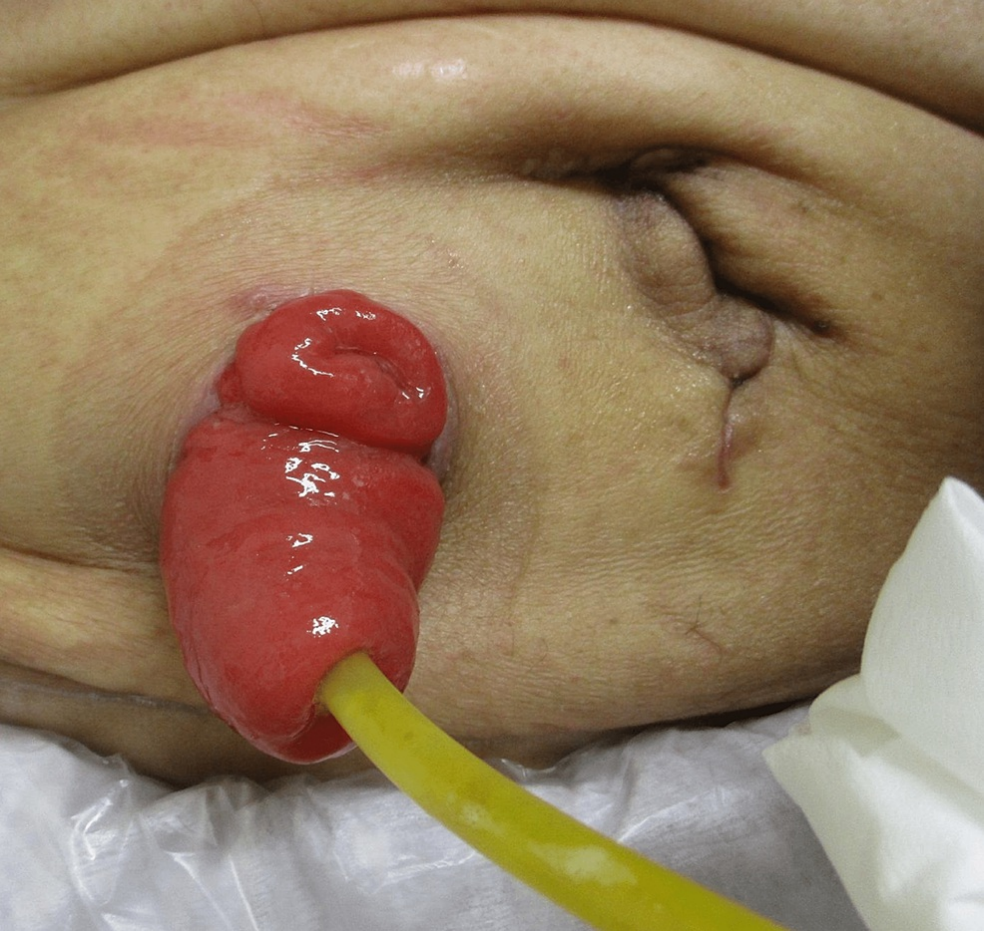
Decompression catheter as a treatment for small bowel obstruction Trans-stomal decompression catheter is inserted through the efferent limb. Image created by the authors

Small bowel obstruction (SBO) frequently occurs in the early postoperative period after colorectal surgery, and its incidence significantly increases to 23% when DI is created [9-11]. SBO at the stoma site is often called stoma outlet obstruction (SOO) or stoma-related obstruction (SRO). Ulcerative colitis, increased operative time, stoma limb rotation, and thick rectus abdominis muscle have been shown to be risk factors. SOO/SRO is diagnosed by excluding SBO other than at the stoma site [12-14] (Figures 5, 6). Local treatment with trans-stomal decompression is most effective (Figure 7), and immediate surgical intervention is rarely required. Early stoma closure may be scheduled in recurrent cases. Preventive techniques have been controversial, and some reports have advocated that the distance between the stoma site and anastomosis must be long enough to avoid tension or kinking [15, 16], whereas some have recommended suture fixation of the stoma to the abdominal wall [17, 18].

High output stoma

The normal output volume of ileostomy varies from 600 to 1,200 ml/day and is influenced by the digestion of liquid and food [19, 20]. When it exceeds the normal limit, dehydration with electrolyte imbalance occurs, and acute and/or chronic renal impairment may be induced. This is called a high output stoma (HOS) and is inconsistently defined in the literature when the output exceeds 2,000ml/day for three or more consecutive days [19], 1,500ml/day for two consecutive days [21], 2,000ml/day [22], or 1,000ml/day for more than three consecutive days [23]. Patients typically present with weight loss, dry mouth, thirst, reduced urinary output, and decreased blood pressure. Laboratory testing typically shows hypomagnesemia and depletion of sodium in the urinary output [20]. The incidence of HOS is approximately 16% [19, 21], and there are multiple risk factors, including short bowel, diabetes, medication (metformin, prokinetic drugs, or sudden withdrawal of steroids or opiates), Clostridium difficile infection, intra-abdominal sepsis, and bowel obstruction [19, 24].

Fluid balance checks and electrolyte monitoring are mandatory for the treatment of HOS. It is necessary to exclude underlying pathologies other than stoma, such as enteritis, intra-abdominal sepsis, or bowel obstruction. Rapid fluid resuscitation is required to prevent acute renal failure due to severe dehydration. The intake of hypotonic fluid (water, coffee, tea, or juice) must be restricted to 500-1,000 ml/day in order to preclude further increases in output [19, 20]. Antimotility drugs (loperamide) are generally used as the initial medication, while antisecretory drugs (H2 antagonists or proton pump inhibitors) are used to reduce gastrointestinal secretion. Hypomagnesemia is treated with oral or intravenous supplementation. Early stoma closure should be considered in refractory cases. The keys to prevention are early recognition and interventions for dehydration. In-hospital and outpatient protocols have been suggested, consisting of output monitoring, guidance for liquid and food intake, and patient interviews using telecommunication tools [25-27]. 

Stomal prolapse

**Figure 8:**
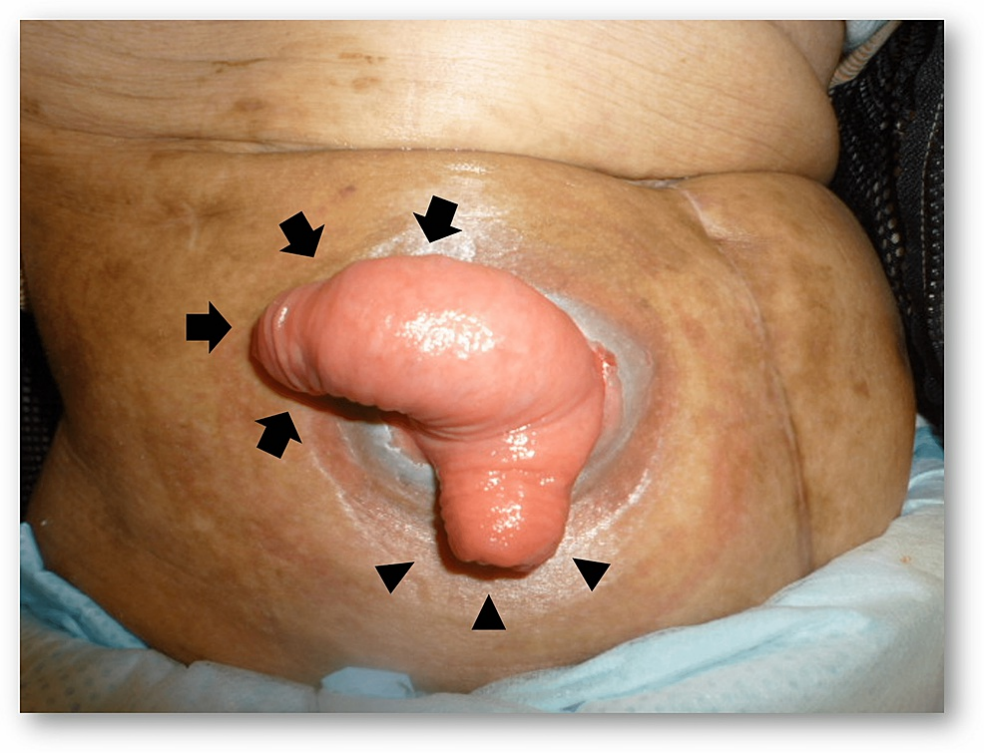
Stomal prolapse Full-thickness protrusion occurs in both the afferent (arrows) and efferent (arrowheads) limb of the diverting ileostomy. Image created by the authors

Stomal prolapse is defined as a full-thickness protrusion of the stoma (Figure 8). Inversely protruding bowel may compromise stoma care. Stomal prolapse occurs in 3-11% of ileostomies [4, 28]. Redundant and mobile stoma limbs and a wide trephine at the stoma site have been proposed as risk factors [29]. When prolapse occurs, a manual reduction is attempted, but its effect is mostly temporary. Reducible prolapse does not require further intervention, whereas non-reducible prolapse often requires painful and durative manual reduction. The slug method is a unique procedure for manual reduction, in which 50% glucose solution is applied to the prolapsed edematous mucosa [30]. Increased peristalsis and fluid exudation from the mucosa assist in manual reduction.

In cases of incarcerated prolapse, emergency surgery is indicated, and the ischemic bowel must be resected. Many surgical techniques have been proposed, including local repair, revision, or relocation with laparotomy [29, 31-33]. Among these procedures, local repair using a stapler device appears to be less invasive, with the lowest recurrence rate of 4% [29]; however, the majority of the reports were retrospective studies with a relatively small number of patients. Therefore, further prospective studies with longer follow-up periods are warranted. Methods for preventing stomal prolapse have not yet been established. 

Parastomal hernia

**Figure 9:**
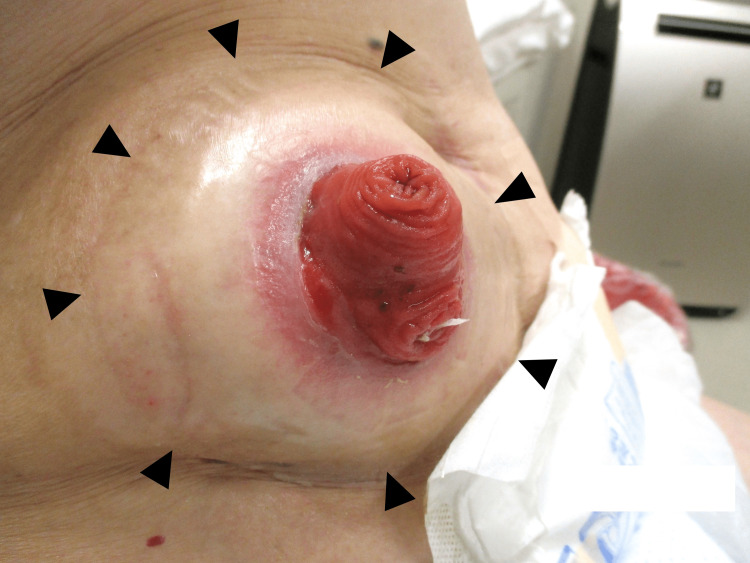
Parastomal hernia with loop ileostomy Bulging of the peristomal area without stomal prolapse (arrowheads). Image created by the authors

**Figure 10:**
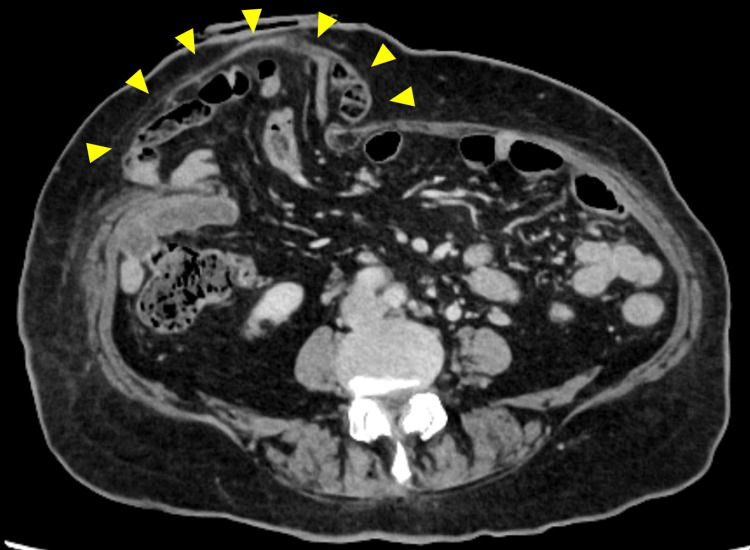
CT image of a parastomal hernia A large area of the bowel and mesentery (arrowheads) are herniated through the stomal orifice outside the abdominal wall. Image created by the authors

A parastomal hernia (PSH) is an incisional hernia of the stoma site (Figures 9, 10). According to the literature, its incidence varies to as high as 48%, depending on the stoma type [4, 28]. Generally, ileostomy has a lower incidence of PSH than colostomy, and the incidence of PSH in loop ileostomy is 1.5% [2]. The symptoms of PSH include pain, peristomal discomfort, and difficulty in fitting pouch appliances, resulting in fecal leakage. Numerous risk factors contribute to the development of PSH, including patient-related factors (obesity, elderly, smoking, use of steroids, constipation, less exercise, chronic obstructive lung disease, etc.) and technical surgical factors (inappropriate stoma site selection, oversizing fascial trephine, emergency surgery, etc.) [32, 34]. 

In the case of temporary ileostomies, such as DI, nonoperative management is recommended with the use of a supporting belt, weight control, and exercise. Surgery is considered for patients presenting with an incarcerated bowel obstruction. Nonoperative management may be a better option for patients with concomitant disease or without complaints, considering the high recurrence rate requiring additional surgery for those who underwent PSH repair [35]. A prophylactic mesh may reduce the incidence of PSH if placed at the time of the index surgery [36]; however, it may not be necessary for DI patients for whom stoma closure is expected shortly after its creation.

## Conclusions

Certain ileostomy complications can be prevented by surgeons during stoma creation. However, inevitable complications do exist due to the nature of ileostomy. Therefore, perioperative patient evaluation and education, in-hospital assessment, and postoperative surveillance protocols provided by a multidisciplinary team are crucial for early recognition and appropriate management of ileostomy complications.
